# Formaldehyde—A Key Monad of the Biomolecular System

**DOI:** 10.3390/life3030486

**Published:** 2013-08-16

**Authors:** Christian R. Noe, Jerome Freissmuth, Peter Richter, Christian Miculka, Bodo Lachmann, Simon Eppacher

**Affiliations:** 1 Department of Medicinal Chemistry, Faculty of Life Science University of Vienna, Althanstr. 14, Vienna 1090, Austria; E-Mail: bodo.lachmann@univie.ac.at; 2 Department of Pharmaceutical Chemistry, University of Frankfurt, Max-von-Laue-Str. 9, Frankfurt D-60438, Germany; E-Mails: jfre@tetragon-chemie.com (J.F.); peter.richter@aqura.de (P.R.); christian.miculka@hotmail.com (C.M.)

**Keywords:** formaldehyde, D-glucose, prebiotic, stereoelectronic effect, helical chirality

## Abstract

Experiments will be presented and reviewed to support the hypothesis that the intrinsic reactivity of formaldehyde may lead to the formation of a rather comprehensive set of defined biomolecules, including D-glucose, thus fostering concepts of evolution considering the existence of a premetabolic system as a primordial step in the generation of life.

## 1. Introduction

The term “monad” has been used by Gottfried Leibniz to describe the “elementary particles”, but actually it has been in use by philosophers since early times starting with the Pythagoreans, and later on by Giordano Bruno and others. The characteristic of a monad is that it does not just define a smallest physical unit from a materialistic point of view, but that is has—above all—a “functional” meaning. One might even be tempted to say that this term seems to break down the universalism debate to its smallest unit: “Materialism *versus* idealism”, “structure and function”, “being and time”, “essentia *et* accedentia” (the terms “accidentia” and “accedentia” are both in use, frequently not sharply separated; the first term denotes the accidental nature, while the latter means that something is joining). The whole dualistic philosophical debate can be held at the level of the monad. Applying such a broad interpretation in our time of systems biology, the old fashioned term seems particularly helpful to define smallest units of a system. Beyond that, these units intrinsically contain the potential for the relevant functional parameters of the system. In the case of the biomolecules, the term monad stands for a smallest biomolecule exhibiting an intrinsic reactivity sufficient to build up a primordial premetabolic system, from which the metabolic system can evolve as one of the crucial functional elements of living systems apart from compartmentalization, homoeostasis and propagation. Over the last three decades, this paper’s corresponding author’s research group has published a number of contributions relating to the hypothesis that the intrinsic reactivity of formaldehyde may lead to the formation of a rather comprehensive set of defined prebiotic biomolecules, thus fostering concepts of evolution considering the existence of a premetabolic system as a primordial step in the generation of life. We will offer a definition of this new term later in this paper. In this article we will summarize our experiments and contributions to supporting the notion that formaldehyde can be considered a key monad to the formation of larger functional biomolecules besides hydrocyanic acid and formamide [1].

## 2. Discussion

### 2.1. From Formaldehyde to Glycolaldehyde, and on to Sugars

Three out of the six most relevant elements of biomolecules (C, H, O, N, P, S) are present in the four-atom molecule formaldehyde. It is also evident that carbohydrates may be seen as oligomers of formaldehyde. Starting with the work of A.M. Butlerov, the formose reaction has been extensively studied both from the aspect of opening up a source for nutrition and to demonstrate the role of formaldehyde in prebiotic chemistry [2,3]. The complexity of the reaction mixture obtained in the formose reaction resembles the complexity of the “Miller-experiment”, which has become the standard reference to demonstrate the formation of biomolecules under prebiotic/abiotic conditions [4,5,6,7]. However, in view of the complexity of the reaction mixtures, both Butlerov’s and Miller’s experiments fail to explain how from such complex mixtures a metabolic system could arise, the formation of which even potentiates complexity. When P. Decker analyzed the complex mixture of the formose reaction for the first time, he could prove that biologically relevant monosaccharides, such as glucose, were formed only in minute amounts [8]. An explanation for the evolution of life from such complex mixtures would—due to the low statistical probability—point to a “universal” uniqueness of life. The application of a *vis vitalis* on the other hand is beyond experimental access. The rise of prebiotic chemistry as a research field trying to break down the seemingly unlimited complexity of potential biomolecules was the consequence, with pioneers such as H.C. Urey, L. Orgel, I. Oro, J.P. Ferris and A. Eschenmoser promoting studies on the role of intrinsic molecular reactivity in particular. The search for pathways of self-constitution of simple molecules capable of existing in a prebiological environment nowadays plays an important role in developing concepts of the prebiotic formation of biomolecules [9,10,11,12,13].

**Figure 1:**
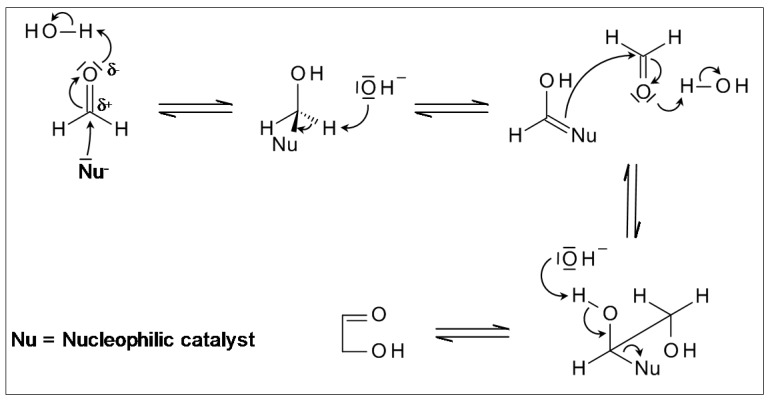
Formation of Glycolaldehyde via *umpolung* from Formaldehyde.

Glycolaldehyde is the simplest sugar, therefore also called diose. Its formation is the initial step of the formose reaction. The compound has also been detected in interstellar clouds [14]. The authors suggested that the typical environment of an interstellar cloud might be favorable to sugar synthesis, since interstellar formaldehyde is ubiquitous. C-C bond formation of two formaldehyde molecules to form glycolaldehyde requires a catalyst. The relevant property of formaldehyde, which allows C-C bond formation, is its ability to undergo an *umpolung* reaction through a reversal of its partial charge (Figure 1). The carbon atom of formaldehyde has a positive partial charge due to the attached oxygen atom. Electronegative partners, such as oxygen in hydroxy groups, nitrogen in amino groups or particularly the carbon atom of a cyano group add easily to this positively charged carbon atom generating sp^3^ hybridisation at the carbon atom and polarizing the C-H bond. Deprotonation can occur, resulting in a carbanion that adds to the positive carbon atom of the reaction partner.

**Figure 2:**
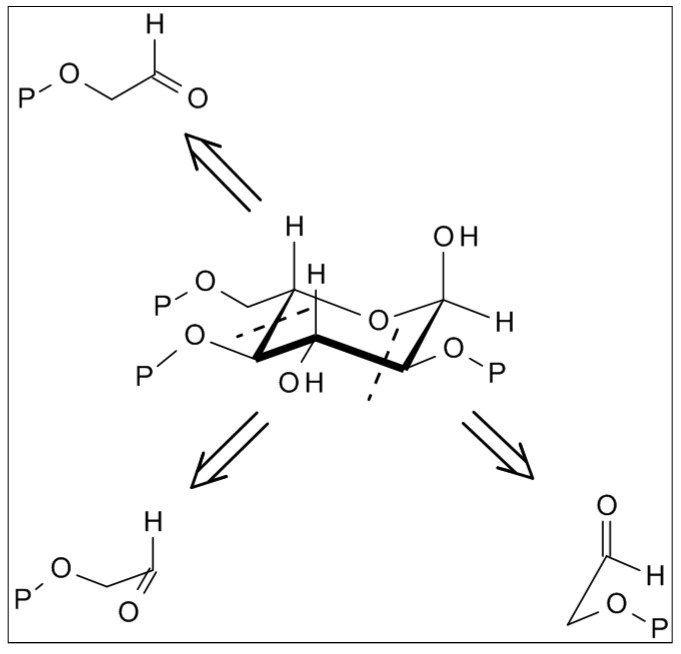
Trimerisation of protected glycolaldehyde (Shown for α-L glucose).

Clay environments or radiation might induce this step as well [14]. Not only is *umpolung* the first step in glycolaldehyde formation from formaldehyde, but also the enabling step toward a whole range of other potential prebiotic pathways for self-constitution of other biomolecules, such as amino acids by addition of ammonia or nitrile groups to formaldehyde [15,16,17]. The retrosynthetic analysis of glucose reveals that the molecule is made up of three units of glycolaldehyde (Figure 2). This determination was first made by E. Fischer, but began to be investigated in a systematic and experimental mannerby our group in Vienna and A. Eschenmoser’s group in Zürich only about 30 years ago [18,19]. Hydroxy-protected gylcolaldehyde was submitted to various aldomerisation conditions, and in spite of different protective groups at the hydroxy group, the results obtained in terms of the distribution of reaction products were almost identical by both groups. 

Somehow surprisingly, the main product in this reaction [19,20,21] was the allose derivative, which is not a dominant sugar component in our environment. Much less surprisingly, the aldomerisation reaction of glycol aldehyde in the presence of formaldehyde led to the predominant formation of pentoses with ribose as the main product [19,22,23]. In all these experiments the complexity of the resulting abiotic sugar mixture was broken down very significantly by use of the hydroxyl-protecting group. While, the preferential formation of ribose corresponded well into the present system of biomolecules, the formation of allose—instead of the expected abundant biomolecule glucose—still left questions open.

### 2.2. “Stereoelectronic Effects”—A Key Contributor to the Amplification of Chirality of Biomolecules

The trimerisation of glycolaldehyde is a demonstration that seemingly complex natural compounds are easily formed in self-constitution reactions. The variety of options for Corrin cyclisation in Vitamin B12 total synthesis was certainly an early impressive example of the applicability of this observation even to synthetic organic chemistry [24,25].

**Figure 3:**
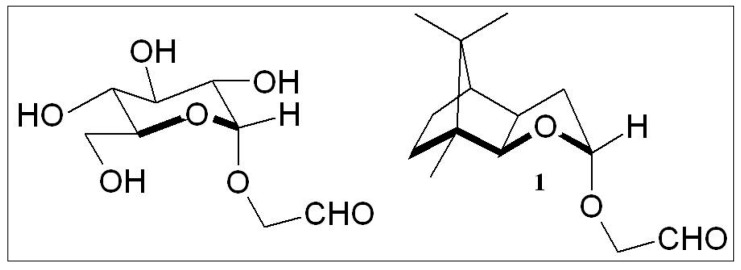
Structural relationship of the glycosidic centers in **1** and a corresponding *O*-glycolaldehyde-α-L-glucopyranose. In both molecules the axial position of the electronegative exo-cyclic *O*-atom is stabilized by the endo-cyclic *O*-atom induced anomeric effect.

The preference of one enantiomer over the other in naturally occurring sugar led us to the obvious question, whether there was a biological selection mechanism for one anantiomer, like D-glucose, later in evolution or whether the self-constitution from formaldehyde might afford the enantiomer directly. We addressed this aspect of chirality experimentally by blocking the hydroxyl group of the formaldehyde dimer, glycol aldehyde, via a glycosidic linkage typical for carbohydrates as shown in Figure 3. A substitution at the oxygen atom of glycol aldehyde is anyway a prerequisite to arrive at a selective course of aldomerisation. The aim was therefore also to investigate, whether a glycosidic linkage might be a suited chirality inducer in aldose formation. For this purpose we designed a clearly non-prebiotic, artificial carbohydrate-terpene chimera. This was achieved by annulating a five or six membered lactol ring to a bornane system in a manner that would allow access of reaction partners only from one side of the ring [26,27,28,29,30], leading to stereoselectivity.

Thus, the reactivity of the glycosidic center of the resulting “terpenoid carbohydrate” could be studied systematically in an organic environment. This approach proved to be very helpful, not only because of solubility reasons, but also because stereoelectronic effects are significantly more effective if the oxygen lone pairs cannot interact with protons in the solvent. With respect to intrinsic molecular reactivity, the conditions in an organic solvent resemble much more those in an interstellar cloud than aqueous conditions, in which there is no solvent at all. Apart from that, the bornane-derived compounds also have been shown to be useful chiral protecting groups for organic synthesis [31,32,33,34,35,36,37]. Stereoelectronic effects are useful to explain influences on the stereochemistry of molecules with respect to their structure, reactivity, or properties beyond traditional bond or steric effects. We have used the bornane-derived model system extensively to investigate these effects of an additional bond stabilisation via n-σ *, σ-σ *; π-σ * or π-π * interactions on the conformation and reactivity [38,39,40]. Although they have been the subjects of studies for decades, we believe that their fundamental role in the chemistry of life still provides ample room for further studies. 

Secondary alkyl-aryl-carbinols exhibited a significant predictive enantiomer-selectivity in acetal formation under reversible conditions with the designed lactols [41,42,43,44]. The effect of enantiomer selectivity was also observed in ether formation [45,46], thioacetal and thioether formation [47] and in 1,4-addition of amines [48,49]. Added steric hindrance led to increased enantiomer selectivities of up to 14:1 [50,51,52]. We stated that as a rule in reactions involving connecting two chiral centers by an electron donating atom (like oxygen or sulphur) of two reaction partners each featuring a bulky substituent (e.g., alkyl), a σ *- or π *-receptor substituent (e.g., aryl or oxygen) and hydrogen at the chiral center, the diastereomer with like relative configuration of the three substituents will be preferentially formed once equilibrium is reached under reversible reaction conditions. [43,44,53]. (We should highlight that this latter definition of relative configuration is a functional one, based on steric and electronic effects, rather than the atomic weight-based Cahn, Ingold, Prelog nomenclature.) Interestingly enough, in the early phases of these reactions stereoselectivity tends to be reversed, *i.e.*, the diastereomer with unlike relative configuration of the three substituents would be preferentially formed, only to equilibrate according to the rule stated above. This was first observed in experiments on thioacetal formation, and later confirmed to apply to other structural classes as well [54,55,56]. Although not yet fully understood, it may be assumed that stereoelectronic effects have an impact also on the kinetics of bond formation.

### 2.3. D-Glucose—A Product of Molecular Self-Constitution

**Figure 4:**
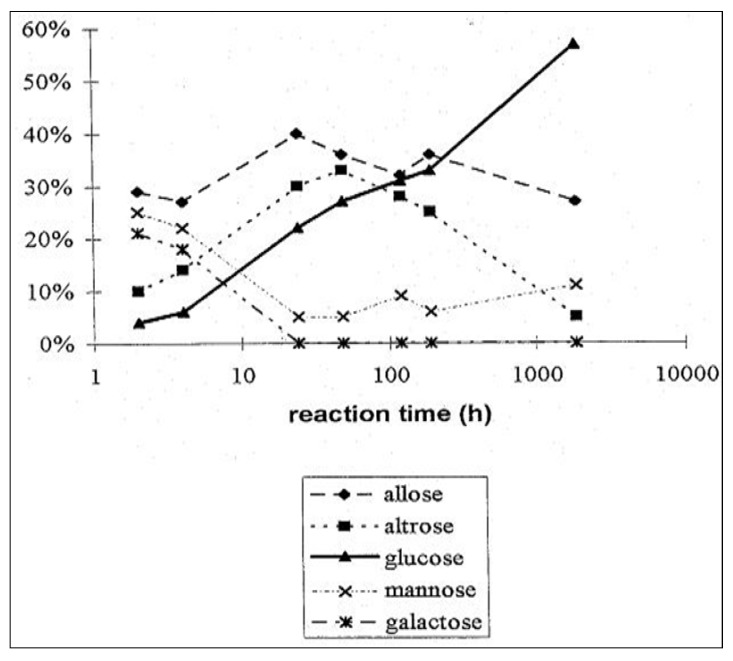
Typical course of an aldomerisation of 1. Only the main products are shown. Reaction conditions: solvent—diethylether (50%·w/w); base sodium hydroxide (0.5 equivalents); room temperature.

The reversal of diastereoselectivity from the starting phase to the equilibrated phase, together with slight inconsistencies in product distribution in the glycolaldehyde trimerisation in our earlier experimental series prompted us to extend the duration the trimerisation reaction in diethylether (50% w/w) with 0.5 equivalents sodium hydroxide at room temperature to observe eventual further changes in product distribution. In fact, a shift was observed away from allose as the predominant aldose after 4 h, which afforded a product distribution after 11 weeks in which glucose (Figure 4) had become the predominant aldose in the trimerisation reaction [22,57,58].

Analogous experiments towards pentose aldomerisation exhibited a similar shift in isomer distribution from predominating ribose to xylose [22,23,58].

**Figure 5:**
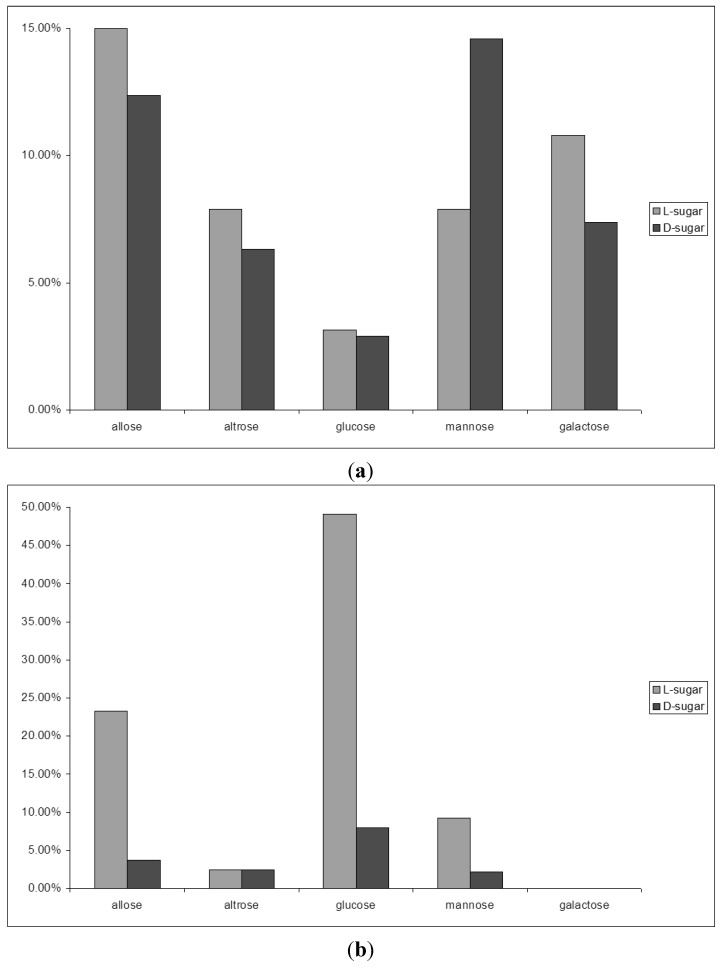
(**a**) Composition of the reaction mixture after 4 h reaction time. Only main products are shown; (**b**) Composition of the reaction mixture after 11 weeks reaction time.

Since the carbohydrate-terpene chimera **1** corresponds to an α-L sugar configuration at the pseudo-anomeric center, we were also able to investigate the propagation of this absolute configuration to the sugar products. Enantioselective capillary electrophoretic methods unequivocally showed that the expected L-glucose became the prevalent aldose, making up 49% of the aldohexose mixture (Figure 5a,b) [57,58,59,60,61]. We chose the L-configurated sugar model to exclude contamination with a D-glucose during the experiments and clearly, the enantiomorph experiment would have led to the D-glucose as the dominant hexose. In an alternative approach, Pizzarello later demonstrated that enantiopure short peptides are able to catalyze the stereospecific formation of tetroses [62] and pentoses [63] from glycolaldehyde.

The analogous experiment in the presence of formaldehyde yielded L-xylose as the predominant product upon prolonged reaction time [22,23,57]. These results demonstrate that stereoelectronic effects via the glycosidic linkage play a key role in the diastereoselective formation of glucose, and the enantioselective formation of one predictable glucose enantiomer from the trimerisation of a chiral glycolaldehyde precursor, pointing to their importance in the amplification of chirality in prebiotic biomolecular evolution.

### 2.4. From Glycolaldehyde Phosphate to RNA

RNA—with its potential both for self-reproduction and catalytic effect—has been considered the crucial “molecule of life” and has therefore been in the focus of prebiotic research of the last decades [64,65]. A. Eschenmoser, by synthesizing potential prebiotic RNA alternatives based on variations of the sugar, nucleobase and phosphate moieties, and determining their functional properties in terms of strand pairing, helix formation and replication, demonstrated that ribose apparently is the only aldose, which intrinsically exhibits the required structural—constitutional, configurational and conformational—features to perform the intended functions [12,66,67]. These experiments allow hypothesizing that even RNA is a product of molecular self-constitution. At this point, it is worth mentioning that stereoelectronic effects play an important role also in double helix formation [40,68]. 

The shift in product distribution from allose to glucose, which was observed in our experiments with hexoses, is in line with the prevalence of these sugars in biological systems. In the pentose experimental series however, xylose dominated over ribose. Xylose frequently occurs in biological systems as well, but it has obviously not been the sugar molecule of prebiotic choice for the nucleic acid backbone. To assess the effect of prolonged reaction times in pentose formation, the experiments were carried out following A. Eschenmoser’s approach by using phosphate as protective group at the hydroxy group of glycolaldehyde. Only a very slow change of isomer distribution could be observed in this case under the basic reaction conditions [22,23,61]. It may be assumed that the phosphate groups on the generated sugars protect them from the base attack required for further product isomerisation. These experiments yielded ribose phosphate as the dominant sugar, supporting the role of glycolaldehyde phosphate as the first nucleotide precursor as proposed by A. Eschenmoser [69,70] in the search for RNA molecular self-constitution [71]. Since nucleobases do not readily add to ribose, Sutherland has proposed an alternative mechanism via arabinose derivatives; however glycolaldehyde remained the assumed starting material [64].

### 2.5. Cyanhydrine Formation as Potential Prebiotic Source of Hydroxy and Amino Acids and Their Polymers

**Figure 6:**
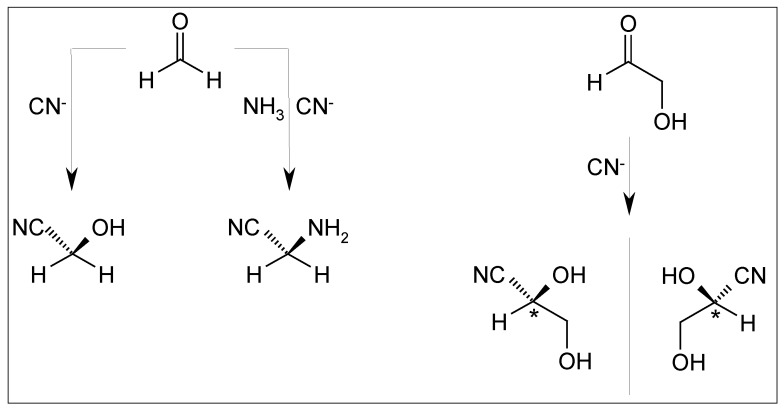
Cyanhydrine formation from formaldehyde and glycolaldehyde.

Cyanohydrine formation via reaction of formaldehyde with the carbon atom of a negatively charged cyano group, and the same reaction involving ammonia, in addition yield the prochiral nitrile of the proto hydroxy acid glycolic acid and the proto amino acid glycine, respectively (Figure 6) [72]. Both compounds have been subject of prebiotic studies.

In the cyanohydrine reaction of glycol aldehyde a chirality center is generated.

The preference in acetal formation of such cyanohydrines is predictable [73]. Hydrolysis of the nitrile group results in α-hydroxy acids [52]. These compounds are bifunctional and may be subject to stereoelectronic control in their reactions. In contrast to acetal or ether formation, the linkage between the two reacting chiral centers in an ester or amide consists of two atoms (O-C=O-). Experiments indicate that stereoelectronic control can influence also the course of the reaction in such cases of higher distance between the chirality centers [74,75,76]. Thus, it may be hypothesized that self recognition of enantiomers might play a role also in oligomerisation and polymerization reactions of hydroxy acids and amino acids in peptide and protein formation.

### 2.6. Back to Formaldehyde: Polyformaldehyde—Centrochirality to Helical Chirality

The only chirality-determining element in biomolecules discussed so far is the tetravalent carbon atom. As described above, experiments have shown that directed amplification of chirality due to the action of stereoelectronic effects might have been an intrinsic element in molecular self-constitution [53]. Further optimisation of enantiomer generation up to enzymatic efficiency may have been either a late step in prebiological evolution or in early biological evolution. However the primordial source for the unique enantiomerically pure tetravalent carbon has remained an open question. 

**Figure 7:**
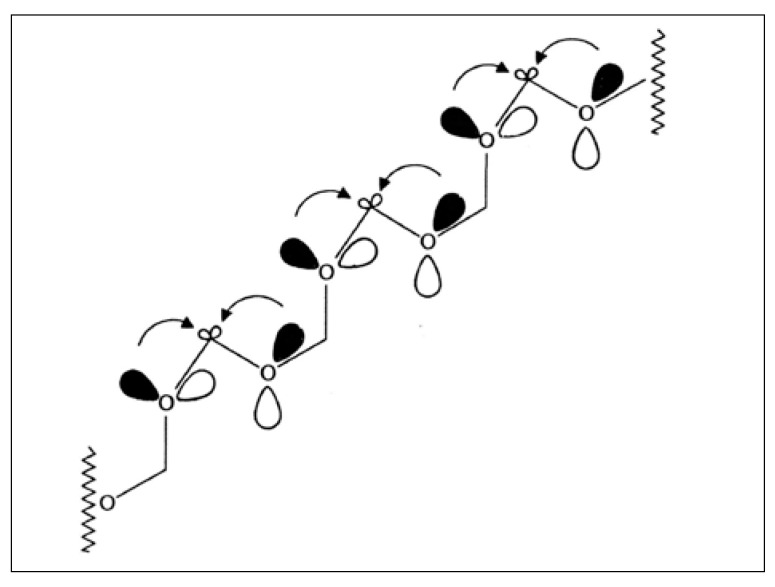
Stabilisation of (polyoxymethylene) due to stereoelectronic effects.

Formaldehyde polymerizes easily to form polyformaldehyde (polyoxymethylene). Due to a continuous sequence of stereoelectronic stabilizations, polyformaldehyde assumes a rather stable helical conformation, either left handed or right handed, thus changing from a prochiral entity into a chiral entity (Figure 7). Oligomers crystallize as conglomerates of crystals made up of either right-handed or left-handed molecular units [77,78]. It could be shown experimentally that a chirality center of the b, pl, H-type will induce helicity into an oxymethylene chain with a predictable handedness [79]. In one experiment significant asymmetric 1,10-induction of chirality was obtained in a Grignard reaction along the oxymethylene chain [80]. Experiments also showed that our diastereoselectivity rule also applied across an oligomeric polyoxymethylene chain [79,81]. In fact the helical conformation of the -O-C-O- sequence in polyformaldehyde resembles rather the atomic arrangement of -O-Si-O- helices in a quartz crystal or atomic arrangements in other inorganic crystals [79]. It may be hypothesized that a specific precipitation or formation of one enantiomer of a polyoxymethylene helix might easily occur in an inorganic chiral environment, thus providing the basis for further amplification of chirality based on stereoelectronic effects. The formation of sugar-silicic acid complexes might play a role in the “inorganic to organic” chirality induction in the self-constitution of aldoses [82]. Although this concept does not explain the origin of chirality in our world in principle, it provides one likely explanation for the transfer of chirality from inorganic to organic matter in prebiotic evolution.

## 3. Conclusions—“From Accedentia to Essentia”

Research on the origin of life has traditionally had a strong focus on the environmental conditions crucial for the “decisive step” in the generation of life. Planetary conditions [83], interstellar clouds, and deep-sea vents have been subject of search for the origin of life. We, and others, have shown and argue that intrinsic molecular properties nonetheless lay the foundation for self-constitution of functional biomolecules when exposed to extrinsic environmental factors. Factors like electronegativity of atoms and their spatial arrangement set by the valence rules play the key role, as do stereoelectronic effects or the rules of ligand field theory.

In line with the philosopher Baruch Spinoza, who had declared that we experience something as accidental when we do not understand its essence/origin [84], we hypothesize that prebiotic evolution of biomolecules was not primarily an accidental process only provoked by specific environmental conditions, but was to a large degree essential due to the intrinsic reactivity of elements and molecules involved. Neither glucose, nor an amino acid nor RNA requires “life” for their formation, nor would their detection prove the existence of life. It may be assumed that intrinsic molecular reactivity has led to a broad but limited set of potential biomolecular structures with constant functional elements—monads—but varying composition depending on the environmental conditions. As soon as such a proto-biomolecular chemical system has arrived at the stage of compartmentalisation [85,86], a new level of distinct individual existence would have been reached, a premetabolic system. At this point, the reaction equilibrium between biomolecules present in the compartment becomes the homoeostasis of this proto-cell. We speculate that in view of Kolb’s concept of emergence of life by a quantity-to-quality transition of abiotic matter [87], this would correspond to a dialectic step leading to a kind of pre-life entities, which are endowed from the beginning with a variable set of molecules with distinct cross-reactivities constituting a kind of premetabolic system. Driven and complemented stepwise by both accidental as well as essential environmental conditions and by known—and maybe unknown—further central features for emergence of life, the proto-metabolic system of primordial life may have evolved.
